# Monitoring ESBL-*Escherichia coli* in Swiss wastewater between November 2021 and November 2022: insights into population carriage

**DOI:** 10.1128/msphere.00760-23

**Published:** 2024-04-12

**Authors:** Sheena Conforti, Aurélie Holschneider, Émile Sylvestre, Timothy R. Julian

**Affiliations:** 1 Eawag, Swiss Federal Institute of Aquatic Science and Technology, Dübendorf, Switzerland; 2 Department of Biosystems Science and Engineering, ETH Zürich, Basel, Switzerland; 3 Swiss Institute of Bioinformatics (SIB), Basel, Switzerland; 4 Swiss Tropical and Public Health Institute, Allschwil, Switzerland; 5 University of Basel, Basel, Switzerland; University of Wisconsin-Madison, Madison, Wisconsin, USA

**Keywords:** ESBL-*E. coli*, wastewater-based epidemiology, Switzerland, antimicrobial resistance, molecular methods

## Abstract

Antimicrobial resistance (AMR) poses a global health threat, causing millions of deaths annually, with expectations of increased impact in the future. Wastewater surveillance offers a cost-effective, non-invasive tool to understand AMR carriage trends within a population. We monitored extended-spectrum β-lactamase producing *Escherichia coli* (ESBL-*E. coli*) weekly in influent wastewater from six wastewater treatment plants (WWTPs) in Switzerland (November 2021 to November 2022) to investigate spatio-temporal variations, explore correlations with environmental variables, develop a predictive model for ESBL-*E. coli* carriage in the community, and detect the most prevalent ESBL-genes. We cultured total and ESBL-*E. coli* in 300 wastewater samples to quantify daily loads and percentage of ESBL-*E. coli*. Additionally, we screened 234 ESBL-*E. coli* isolates using molecular methods for the presence of 18 ESBL-gene families. We found a population-weighted mean percentage of ESBL-*E. coli* of 1.9% (95% confidence interval: 1.8–2%) across all sites and weeks, which can inform ESBL-*E. coli* carriage. Concentrations of ESBL-*E. coli* varied across WWTPs and time, with higher values observed in WWTPs serving larger populations. Recent precipitations (previous 24/96 h) showed no significant association with ESBL-*E. coli*, while temperature occasionally had a moderate impact (*P* < 0.05, correlation coefficients approximately 0.40) in some locations. We identified *bla*_CTX-M-1_, *bla*_CTX-M-9_, and *bla*_TEM_ as the predominant ESBL-gene families. Our study demonstrates that wastewater-based surveillance of culturable ESBL-*E. coli* provides insights into AMR trends in Switzerland and may also inform resistance. These findings establish a foundation for long term, nationally established monitoring protocols and provide information that may help inform targeted public health interventions.

**IMPORTANCE:**

Antimicrobial resistance (AMR) is a global health threat and is commonly monitored in clinical settings, given its association with the risk of antimicrobial-resistant infections. Nevertheless, tracking AMR within a community proves challenging due to the substantial sample size required for a representative population, along with high associated costs and privacy concerns. By investigating high resolution temporal and geographic trends in extended-spectrum beta-lactamase producing *Escherichia coli* in wastewater, we provide an alternative approach to monitor AMR dynamics, distinct from the conventional clinical settings focus. Through this approach, we develop a mechanistic model, shedding light on the relationship between wastewater indicators and AMR carriage in the population. This perspective contributes valuable insights into trends of AMR carriage, emphasizing the importance of wastewater surveillance in informing effective public health interventions.

## INTRODUCTION

Antimicrobial resistance (AMR) is listed as one of the top 10 most serious global health threats by the World Health Organization (https://www.who.int/news-room/spotlight/ten-threats-to-global-health-in-2019). In 2019, an estimated five million deaths globally were associated with AMR infections (1). Among the factors contributing to these fatalities is the transmission of AMR, with environmental pathways playing a significant role in both the spread of antibiotic-resistant bacteria (ARB) and the acquisition of antibiotic-resistant genes (ARGs) by clinically relevant pathogens (2).

Extended-spectrum β-lactamase-producing *Escherichia coli* (ESBL-*E. coli*) are resistant to clinically important antimicrobial drugs and are considered important vectors in the transmission of ARGs (3). ESBL-*E. coli* are transmissible through human and animal fecal pollution and are commonly found in inflow wastewater worldwide (4–7). Wastewater monitoring provides a comprehensive perspective of AMR carried across a human population (8, 9), and it is more advantageous than the current patient sampling since it avoids privacy and ethical concerns related to the collection of patient data (10). A centralized monitoring of AMR in wastewater can reflect community-level dissemination of AMR, providing an understanding of the evolution and the spread of clinically relevant ARBs (10). A previous study on carbapenemase-producing *E. coli* (CPE) conducted in Dutch municipal wastewater showed its sensitivity in estimating CPE prevalence in humans (11), suggesting that wastewater monitoring can inform prevalence rates and dynamics of other fecally shed antimicrobial-resistant bacteria such as ESBL-*E. coli*.

Recently, ESBL-producing *Enterobacteriaceae* were monitored on a monthly scale in the municipal wastewater of Basel, Switzerland (12). Building on this, a centralized wastewater monitoring system of AMR and ARBs in the Swiss population may enable public health officials to better understand challenges associated with antimicrobial resistance and to target interventions more effectively. Previous studies showed that antibiotic resistance prevalence in bacteria from wastewater follows similar trends to the rates monitored in clinical environments (4, 5, 7, 13).

Trends in ESBL-*E. coli* prevalence in wastewater are likely reflective of trends in ESBL-*E. coli* carriage in the population served. That is, concentrations of ESBL-*E. coli* in wastewater are a function of the prevalence of people carrying ESBL-*E. coli* in the catchment*,* as well as the proportion of ESBL-*E. coli* among all *E. coli* shed by carriers. However, concentrations may also be influenced by sewer fate and transport processes, including temperature-dependent decay and potential non-human sources of ESBL-*E. coli* entering combined sewers from urban water run-off (14). Temporal trends in ESBL-*E. coli* concentrations may therefore reflect changes in carriage within the population, changes in shedding load dynamics, or environmental impacts on fate and transport. Previous studies of ESBL-*E. coli* prevalence report both significant variations in ESBL-*E. coli* prevalence between months (12, 15) as well as relatively stable percentages over time (3, 5, 16, 17).

In this 1-year longitudinal study, we monitored ESBL-*E. coli* in Swiss municipal wastewater weekly from six wastewater treatment plants (WWTPs). Using culture-based methods, we investigated national spatio-temporal variations of ESBL-*E. coli*, developed a model aimed at approximating the prevalence of ESBL-*E. coli* carriers within the community, explored ESBL-*E. coli* correlations with environmental variables, and assessed monitoring frequency impact on ESBL-*E. coli* concentrations estimation. Molecular methods characterized the presence of ESBL genes in 234 isolates. Our findings offer insights into ESBL-*E. coli* prevalence, distribution, and associated genes in Swiss wastewater, and may act as a basis for long-term wastewater-based surveillance of AMR.

## MATERIALS AND METHODS

### Sample collection

**Fig 1:**
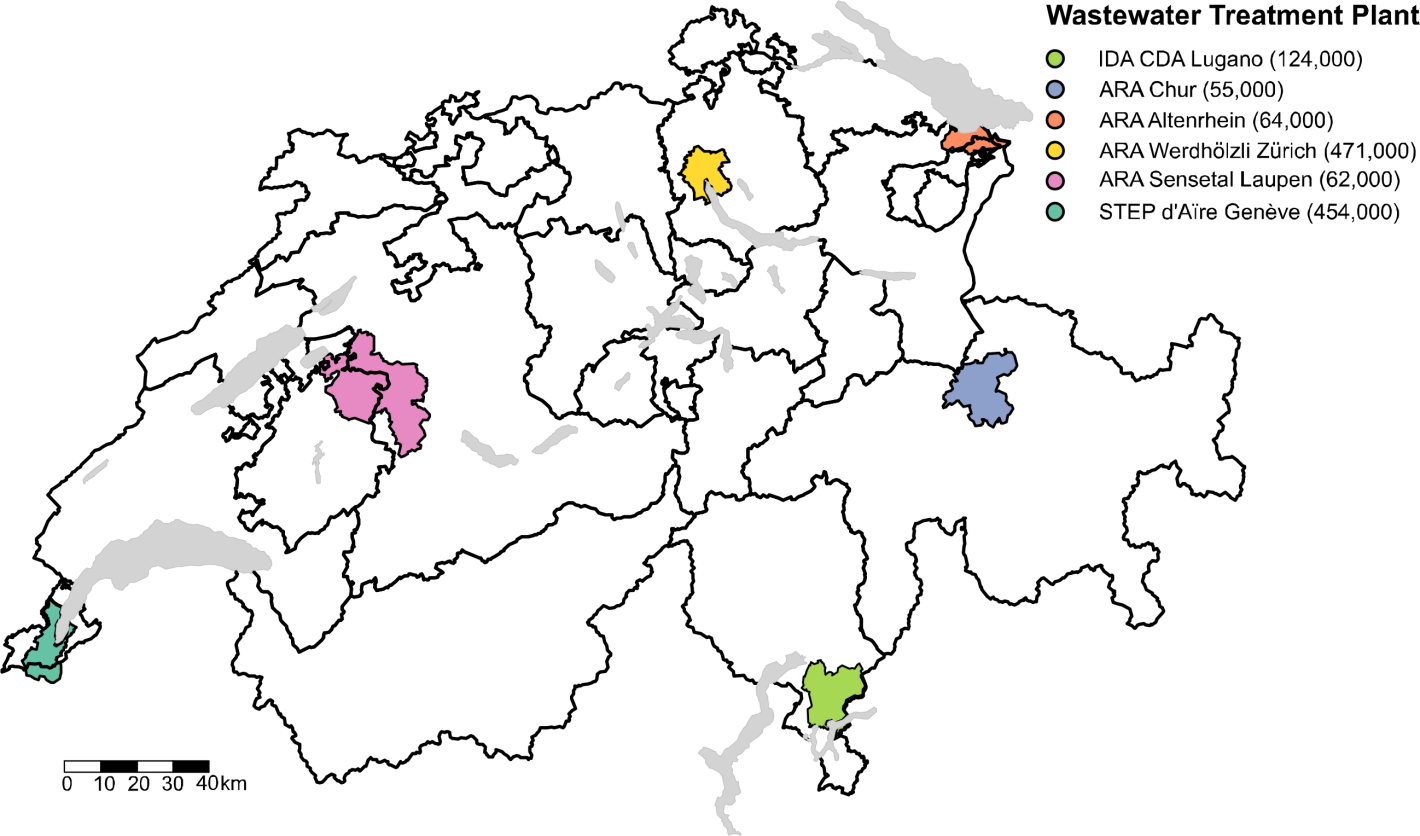
Geographic distribution, catchment area, and number of people served at each of the six investigated wastewater treatment plants in Switzerland. The map was generated in R (v4.1.1) and modified in Inkscape (v1.1.1).

Wastewater samples were collected from six wastewater treatment plants (WWTPs) in Switzerland serving an estimated 1.23 million residents (14% of the population): ARA Altenrhein (64,000 residents), ARA Chur (55,000 residents), STEP d’Aïre Genève (454,000 residents), ARA Sensetal Laupen (62,000 residents), IDA CDA Lugano (124,000 residents), and ARA Werdhölzli Zürich (471,000 residents) (Fig. 1). Samples were collected weekly over a 1-year period from 16 November 2021, to 29 November 2022 (Table S1). Wastewater was sampled using 24-h flow-proportional composite sampling and transported to Eawag (Dübendorf, CH) in high-density polyethylene bottles with ice packs, stored at 4°C, and processed within 48 h.

### Enumeration of total and ESBL-*E. coli*

To enumerate total *E. coli*, wastewater samples were serially diluted 100-fold with sterile 0.9% NaCl and 100 µL were plated on CHROMagar Orientation chromogenic media (CHROMagar, bio-Mérieux, Marcy-l’Étoile, France). To enumerate ESBL-*E. coli*, 100 µL of undiluted wastewater samples was plated on CHROMagar ESBL chromogenic media (CHROMagar ESBL, bio-Mérieux, Marcy-l’Étoile, France). Samples were plated in single replicates until 8 February 2022, and afterward in duplicates. Plates were incubated at 37°C for 24 h. Then, colony concentrations were determined by counting the dark pink to reddish colonies on the CHROMagar Orientation for total *E. coli* and CHROMagar ESBL for ESBL-*E. coli*.

### Isolation, culturing, and DNA extraction of ESBL-*E. coli*

A subset of presumptive ESBL-*E. coli* colonies were isolated every 4 weeks from CHROMagar ESBL plates. Specifically, three colonies per WWTP were streaked on LB Agar (Lennox) and incubated at 37°C for 20–24 h. Single colonies were liquid cultured in Luria Broth (AppliChem), and DNA was extracted from 100 µL of enriched liquid culture by boiling it at 100°C for 1 h. DNA was stored at −20°C for subsequent analyses.

### Screening of ESBL-genes in ESBL-*E. coli* using dMLA

A total of 234 ESBL-*E. coli* isolates underwent screening for 18 ESBL-gene families using the digital Multiplex Ligation Assay (dMLA) (18). All isolates were analyzed in three separate 96-well plate reactions; each set of reactions included three no template controls (NTC) with DNA-/RNAse free water and three positive controls with one DNA artificial template complementary to 1 of the 36 probe-pairs. The 72 half-probes used in the dMLA were mixed to achieve a final concentration of 1 µM each. Subsequent ligation and PCR followed the previously described method (19) and were carried out in 96-well PCR Skirted plates (Milian) sealed with SilverSeal Sealer (Greiner Bio-One GmbH). Each well contained only one isolate or control sample. All PCR products (4 µL from each), barcoded during the dMLA protocol, were pooled and purified using the Wizard Genomic DNA Purification Kit. The pooled purified PCR products were then sent for Illumina MiSeq sequencing (Eurofins Genomics, GmbH, Ebersberg, Germany) with 150 bp paired-end reads.

### dMLA sequence data analysis

NGS reads in FASTQ format were processed using Nextflow (Seqera Labs, v21.10.0.5640). The nsearch software (19) filtered reads with a minimum identity threshold of 0.8 and allowed one expected error per base pair. Subsequently, the IUCON code was implemented in Python (v3.9.2) to expand the degenerated sequences of the filtered reads. The expanded sequences were converted to the correct orientation, and only those matching the target probe-pairs were retained, while the others were discarded. The reads matching the targets were classified according to the forward primer barcode and the target probe-pair, and finally target quantification was carried out in R (v4.1.1).

The number of ESBL-genes in each ESBL-*E. coli* isolate was quantified based on the results of the dMLA assay, using the target counts obtained from sequencing. To determine the detection limits for dMLA, false positive signals from the three NTC samples in each screening were analyzed as previously reported (19). For each probe target, the mean and standard deviation of the number of unique molecules from false positive signals (present in NTC and positive control samples) were calculated independently. The detection limit was determined as the sum of the mean and three times the standard deviation, such that there would be fewer than <0.3% false positives, assuming a normal distribution. Wells in which the number of unique molecules was above the threshold were considered positive for the presence of the corresponding ESBL gene. An additional criterion was that genes were only considered present if detectable by both probe-pairs targeting a single gene.

### Statistical analyses

All analyses were performed in R (v4.1.1) and R Studio (v2022.12.0.353). The percentage of ESBL-*E. coli* over total *E. coli* was calculated by dividing the number of colony-forming units (CFUs) of ESBL-*E. coli* counted on CHROMagar ESBL plates by the number of CFUs of total *E. coli* counted on CHROMagar Orientation plates. The loads (CFUs/(person-day)) of total- and ESBL-*E. coli* were calculated by normalizing estimated concentrations by the corresponding daily flow rate (m^3^/day) of the WWTP and the number of residents connected to the WWTP. For both the percentage of ESBL-*E. coli* over total *E. coli* and the loads, the average was computed when sample duplicates were available to both display and analyze the data. Statistical analysis used Kruskal-Wallis non-parametric tests with Dunn’s pairwise comparisons and Bonferroni adjustment to assess differences in ESBL-*E. coli* percentages and loads among WWTPs and across months within each WWTP. To evaluate the consistency between replicate measurements, Pearson correlation analyses were performed for both total *E. coli* and ESBL-*E. coli* counts.

The Spearman’s correlation coefficient was employed to assess the relationship between ESBL-*E. coli* percentage, total- and ESBL-*E. coli* loads, and the corresponding environmental variables (precipitation, temperature). An alpha value of 0.05 was used to determine the significance of associations, with *P* values corrected using the Bonferroni adjustment. Precipitation data (daily total and cumulative sum over 96 h) and air temperature data (daily average from 6 to 18 UTC) were obtained from the Federal Office of Meteorology and Climatology MeteoSwiss (IDAWEB 1.3.5.0 2016MeteoSwiss).

The impact of monitoring frequency on estimating the mean ESBL*-E. coli* percentage was assessed using a Bayesian inference method (20). Candidate models, gamma and log-normal distributions, were selected to describe temporal variations in ESBL-*E. coli* percentage, and their fit was compared using the Bayesian Deviance Information Criterion. In comparing candidate models, the gamma distribution generally provided a better fit than the log-normal distribution at most sites, leading to its selection for evaluating percentages (Table S2). The standard error of the mean (SE) of the gamma distribution was calculated as $SE=\sqrt{\frac{\alpha }{\beta^{2}\;N}}$, where α represents the shape parameter, β the rate parameter, and N the sample size. Subsequently, the 95% confidence interval (CI) of the mean, indicating the uncertainty of estimates, was calculated using the *z*-distribution.

ESBL-*E. coli* percentage in wastewater is conceptually related to the prevalence of ESBL-*E. coli* carriers within the domestic population contributing to the wastewater. The prevalence of ESBL-*E. coli* carriers within the domestic population is defined as the number of ESBL-*E. coli* carriers in the population (*N*_*C*_) divided by the total population (*N*_*T*_). The prevalence of ESBL-*E. coli* carriers is estimated by the ESBL-*E. coli* percentage in wastewater, denoted as $\pi_{WW}$, divided by the average proportion of ESBL-*E. coli* relative to the total *E. coli* shed only by ESBL-*E. coli* carriers in the population, denoted as ${\bar{\pi }}_{C}$ (equation 1). That is, ${\bar{\pi }}_{C}$ represents the proportion of ESBL-*E. coli* out of total *E. coli* in the gut of ESBL-*E. coli* carriers. In this study, ${\bar{\pi }}_{C}$ is not quantified, so the range of prevalence rates is modeled as a function of ${\bar{\pi }}_{WW}$.


 (1) $$\frac{N_{C}}{N_{T}}=\frac{{\bar{\pi }}_{WW}}{{\bar{\pi }}_{C}}$$

This model assumes constant ${\bar{\pi }}_{C}$, *N*_*C*_, and *N*_*T*_ over the data collection period for estimating ${\bar{\pi }}_{WW}$. *N*_*T*_ includes only people colonized with *E. coli*, so is less than or equal to the total catchment population, considering those who do not shed *E. coli*. The model neglects fate and transport processes within the sewer system and during sample storage and transport. ${\bar{\pi }}_{WW}$ and ${\bar{\pi }}_{C}$ are estimated using the arithmetic mean. The model was applied for each WWTP, where ${\bar{\pi }}_{WW}$ and its 95% CI were predicted from the gamma distribution (see above) and for combined WWTPs, using a population-weighted mean. The 95% CI was computed using the SE of the weighted mean, calculated using the method proposed by Endlich et al. (21), and the *z*-distribution.

## RESULTS

**Fig 2:**
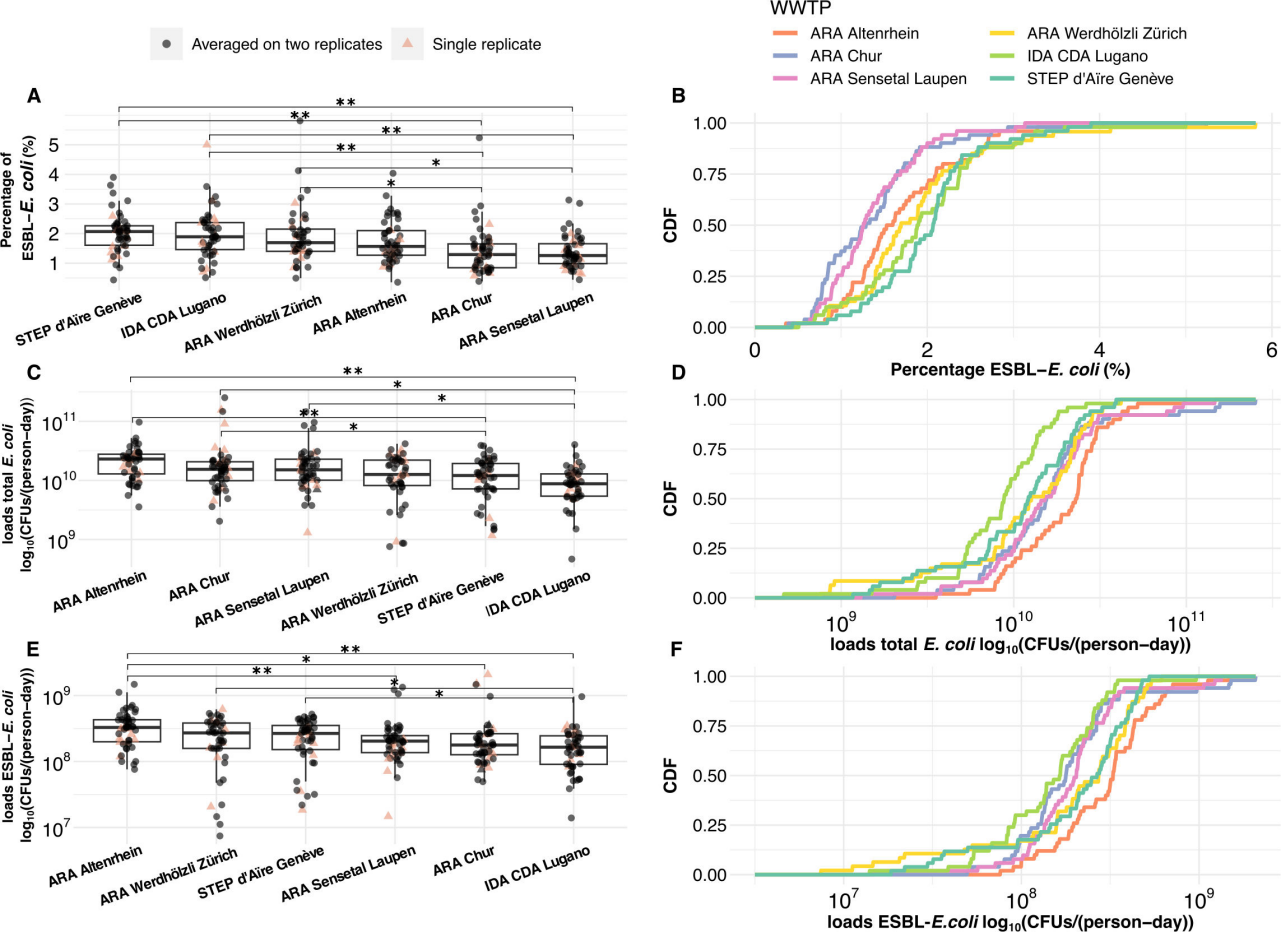
Comparative analysis of percentage and loads of extended-spectrum β-lactamase-producing *Escherichia coli* isolates among six wastewater treatment plants in Switzerland from November 2021 to November 2022. CDF: cumulative distribution function; CFUs: colony forming units; *E. coli: Escherichia coli*; ESBL: extended-spectrum β-lactamase; WWTP: wastewater treatment plant. (**A**) The percentage of ESBL-*E. coli* over total *E. coli* by WWTP. (**B**) The CDF of the percentage of ESBL-*E. coli* over total *E. coli* in each WWTP. (**C**) The log_10_ transformed loads of total *E. coli* by WWTP, and (**D**) the CDF of the log_10_ transformed loads of total *E. coli* in each WWTP. (**E**) The log_10_ transformed loads of ESBL-*E. coli* by WWTP, and (**F**) shows the CDF of the log_10_ transformed ESBL-*E. coli* loads in each WWTP. In panels **A**, **C**, and **E** black circles represent the average of duplicate values, while salmon triangles represent a single value where duplicates were unavailable. * indicates a significant difference with *P* < 0.02 and ** with *P* < 0.001.

Total *E. coli* were detectable in all samples with median load value of 1.4 × 10^10^ (IQR = 1.4 × 10^10^) CFUs/(person-day). Similarly, ESBL-*E. coli* were always detectable with median 2.1 × 10^8^ (IQR = 1.9 × 10^8^) CFUs/(person-day). Consistency between replicate measurements was indicated by the Pearson correlation coefficient (ρ), which showed strong correlations for both total *E. coli* (ρ = 0.97) and ESBL-*E. coli* (ρ = 0.97) counts. The ESBL-*E. coli* median percentage was 1.6% (IQR = 0.89%)*,* and the population-adjusted averaged ESBL-*E. coli* percentage was 1.9% (± 95% CI = 0.1%). More information on summary statistics can be seen in Table S3. ESBL-*E. coli* percentages significantly differed among WWTPs (*P* < 0.001), with the highest median percentage in Geneva (2.1%; IQR = 1.0%) and the lowest in Sensetal-Laupen (1.3%; IQR = 0.7%) (Fig. 2). No significant monthly variations in ESBL-*E. coli* percentages were detected within any WWTPs (Fig. S1; Tables S4 and S5). Total *E. coli* loads differed significantly among WWTPs (*P* < 0.001), with the highest median loads in Altenrhein (2.3 × 10^10^ CFUs/person-day; IQR = 1.4 × 10^10^), and the lowest in Lugano (8.8 × 10^9^ CFUs/person-day; IQR = 4.6 × 10^9^) (Fig. 2). Monthly variations in total *E. coli* loads were observed in Zurich (*P* < 0.001), Sensetal-Laupen (*P* < 0.05), Geneva (*P* < 0.001), and Altenrhein (*P* < 0.05). ESBL-*E. coli* loads varied significantly among WWTPs (*P* < 0.001), with the highest median loads in Altenrhein (3.3 × 10^8^ CFUs/person-day; IQR = 2.3 × 10^8^), and the lowest in Lugano (1.7 × 10^8^ CFUs/person-day; IQR = 1.6 × 10^8^), mirroring the pattern observed for total *E. coli* loads (Fig. 2). Monthly variations in ESBL-*E. coli* loads were observed within Zurich (*P* < 0.001), Sensetal-Laupen (*P* < 0.001), and Geneva (*P* < 0.001). Notably, STEP d’Aïre Genève had significantly higher ESBL-*E. coli* loads in November 2022 compared to November 2021 (*P* < 0.05) and March 2022 (*P* < 0.05).

**Fig 3:**
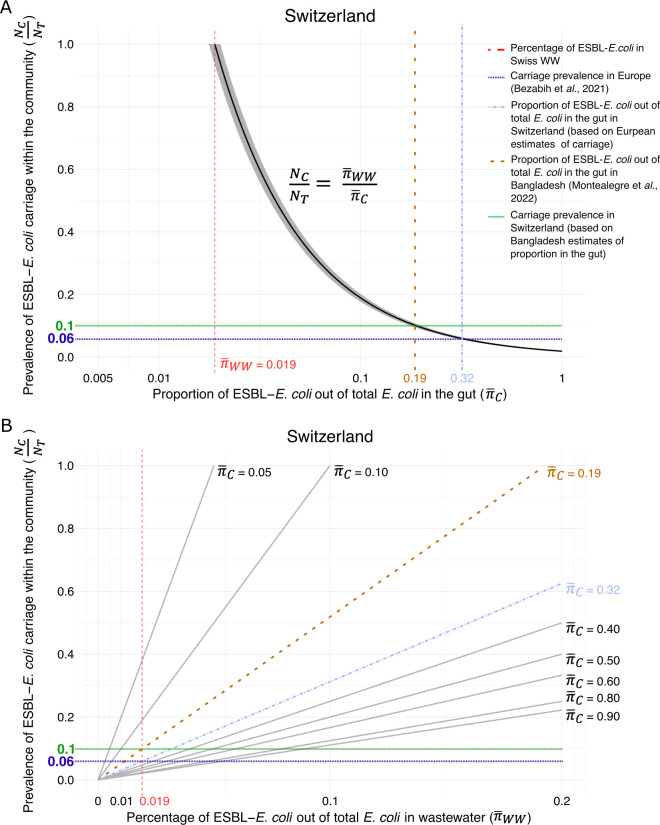
Relationship between percentage of extended-spectrum β-lactamase-producing *Escherichia coli* isolates in wastewater, prevalence of carriage within the community and proportion in the gut in Switzerland. *E. coli: Escherichia coli;* ESBL: extended-spectrum β-lactamase; $N_{C}$: number of ESBL-*E. coli* carriers; $N_{T}$: total population size; ${\bar{\pi }}_{WW}$: mean percentage of ESBL-*E. coli* in wastewater; ${\bar{\pi }}_{C}$: mean proportion of ESBL-*E. coli* relative to total *E. coli* in the gut discharged by carriers into wastewater. (**A**) Visualization of the relationship between percentage of ESBL-*E. coli* detected in wastewater (${\bar{\pi }}_{WW}$) and the estimated community carriage prevalence of ESBL-*E. coli*, based on the assumption that the proportion of ESBL-*E. coli* relative to total *E. coli* in the gut (${\bar{\pi }}_{C}$) is equivalent to the observed percentage of ESBL-*E. coli* in wastewater. The *x*-axis represents various estimates of ${\bar{\pi }}_{C}$, illustrating how changes in this gut flora proportion could influence the inferred prevalence of ESBL-*E. coli* carriage within the community. If the proportion of ESBL-*E. coli* in the gut (${\bar{\pi }}_{C}$) equals the percentage of ESBL-*E. coli* in wastewater (${\bar{\pi }}_{WW}$), then 100% of the community shedding *E. coli* carries ESBL-*E. coli* ($\frac{N_{C}}{N_{T}}$, *y*-axis). The grey-shaded area on the graph represents the 95% CI of the population-weighted mean percentage of ESBL-*E. coli* in wastewater. (**B**) describes the same relationship based on the percentage of ESBL-*E. coli* in wastewater (${\bar{\pi }}_{WW}$) as a predictor (*x*-axis). Here, multiple values of ${\bar{\pi }}_{C}$ are assumed (straight lines), and the prevalence of ESBL-*E. coli* carriage within the community ($\frac{N_{C}}{N_{T}}$, *y*-axis) can be inferred based on the percentage of ESBL-*E. coli* in wastewater (${\bar{\pi }}_{WW}$). The dark-blue dotted line (6%) reflects an estimation by Bezabih et al. (22, 23) for ESBL-*E. coli* carriage within Europe. Assuming this estimation holds for Switzerland, it corresponds to a 32% ESBL-*E. coli* proportion in the gut (light-blue dotted line). Alternatively, if the ESBL-*E. coli* proportion in the gut is approximately 19% (brown dotted line), as estimated in children from Bangladesh (24), then the carriage prevalence in Switzerland is approximately 10% (green line).

We present a mechanistic model that suggests a potential relationship between estimated ESBL-*E. coli* percentage in wastewater and the average percentage of ESBL-*E. coli* in carriers' guts (Fig. 3). The estimated percentage of ESBL-*E. coli* in wastewater provides bounds on the prevalence of ESBL-*E. coli* carriage in the community, based on the average percentage of ESBL-*E. coli* in feces. Considering the observed 1.9% population-averaged ESBL-*E. coli* percentage in Swiss wastewater, the lower bound of prevalence would be 1.9%, which would reflect that all ESBL-*E. coli* carriers shed 100% ESBL-*E. coli*. The upper bound of prevalence would be 100%, which would reflect that 1.9% of all *E. coli* shed by carriers is ESBL-*E. coli*. Using the baseline ESBL-*E. coli* prevalence of 6%, a figure reflective of the broader European context (22), we estimate that about 32% of *E. coli* shed by carriers in Switzerland could be ESBL-*E. coli*. Notably, if we consider the average ESBL-*E. coli* proportion in the gut of children in Bangladesh (19.3%) (23), estimated ESBL-*E. coli* carriage prevalence across all the locations in Switzerland varies between 9.2% and 10.5% (Table 1). Assuming a consistent ESBL-*E. coli* proportion in carriers, observed differences in ESBL-*E. coli* percentages among WWTPs would imply varying carriage rates. Estimated carriage rates range from 7.1% (95% CI = 6.3–7.9%) in Sensetal-Laupen to 10.5% (95% CI = 9.5–11.4%) in Geneva (Fig. S2 and S3).

**TABLE 1:** Relationship between prevalence of ESBL-*E. coli* carriers and ESBL-*E. coli* percentage in wastewater^*a*^

Average proportion of ESBL-*E. coli* out of total *E. coli* in the gut of carriers (%)	Prevalence of ESBL-*E. coli* carriage within the community (%) (95% CI of the mean)						
	Switzerland	ARA Werdhölzli Zürich	STEP d’Aïre Genève	IDA CDA Lugano	ARA Altenrhein	ARA Sensetal Laupen	ARA Chur
1.9	100 (93.8–100)	100 (87.4–100)	100 (96.8–100)	100 (91.8–100)	90.7 (80.4–100)	72.3 (64.3–80.3)	73.5 (62.1–85)
10	18.9 (17.7–20.2)	19.1 (16.6–21.6)	20.2 (18.4–22)	19.7 (17.4–22)	17.2 (15.3–19.2)	13.7 (12.2–15.2)	14 (11.8–16.1)
19.3^*b*^	9.9 (9.2–10.5)	9.9 (8.6–11.2)	10.5 (9.5–11.4)	10.2 (9–11.4)	8.9 (7.9–10)	7.1 (6.3–7.9)	7.3 (6.1–8.4)
30	6.3 (5.9–6.7)	6.4 (5.5–7.2)	6.7 (6.1–7.3)	6.6 (5.8–7.3)	5.7 (5.1–6.4)	4.6 (4.1–5.1)	4.7 (3.9–5.4)
40	4.8 (4.4–5.1)	4.8 (4.2–5.4)	5.1 (4.6–5.5)	4.9 (4.4–5.5)	4.3 (3.3–4.8)	3.4 (3.1–3.8)	3.5 (3 - 4)
50	3.8 (3.6–4.1)	3.8 (3.3–4.3)	4 (3.7–4.4)	3.9 (3.56–4.4)	3.4 (3.1–3.8)	2.8 (2.4–3.1)	2.8 (2.4–3.2)
60	3.2 (3–3.4)	3.2 (2.8–3.6)	3.4 (3.1–3.7)	3.3 (2.9–3.7)	2.9 (2.5–3.2)	2.3 (2.1–2.5)	2.3 (2–2.7)
70	2.7 (2.5–2.9)	2.7 (2.4–3.1)	2.9 (2.6–3.1)	2.8 (2.5–3.1)	2.5 (2.2–2.7)	2 (1.8–2.2)	2 (1.7–2.3)
80	2.4 (2.2–2.5)	2.4 (2.1–2.7)	2.5 (2.3–2.8)	2.5 (2.2–2.8)	2.2 (1.9–2.4)	1.7 (1.5–1.9)	1.8 (1.5–2)
90	2.1 (2–2.3)	2.1 (1.9–2.4)	2.2 (2–2.4)	2.2 (1.9–2.4)	1.9 (1.7–2.1)	1.5 (1.4–1.7)	1.6 (1.3–1.8)
100	1.9 (1.8–2)	1.9 (1.7–2.2)	2 (1.8–2.2)	2 (1.7–2.2)	1.7 (1.5–1.9)	1.4 (1.2–1.5)	1.4 (1.2–1.6)

^*a*^ The table displays the estimated ESBL-*E. coli* carriage prevalence in the community as a function of the ESBL-*E. coli* proportion in the gut and the ESBL-*E. coli* percentage in wastewater. To describe the relationship within each wastewater treatment plant, the gamma arithmetic mean of the wastewater percentage was used. To describe the relationship across all WWTPs (Switzerland), the population-weighted mean was used.

^*b*^ Expected average proportion of ESBL-*E. coli* out of total *E. coli* in the gut in Switzerland is 19.3%; estimate obtained from the mean proportion found in children in Bangladesh in a previous cohort (24).

The model of the relationships between prevalence and percentage of ESBL-*E. coli* in wastewater assumes fate and transport processes are negligible. Investigating the impacts of environmental variables of precipitation and temperature, which might influence fate and transport, showed weak correlations (Spearman’s ρ ranging from −0.36 to 0.45) with ESBL-*E. coli* prevalence in wastewater. Air temperature showed positive correlations with ESBL-*E. coli* percentage in Chur (ρ = 0.45, *P* < 0.01) and Altenrhein (ρ = 0.4, *P* < 0.05), as well as with ESBL-*E. coli* loads in Altenrhein (ρ = 0.41, *P* < 0.05) and Geneva (ρ = 0.43, *P* < 0.05). Precipitation did not significantly influence ESBL-*E. coli* or total *E. coli* loads (Table S6; Fig. S4).

**Fig 4:**
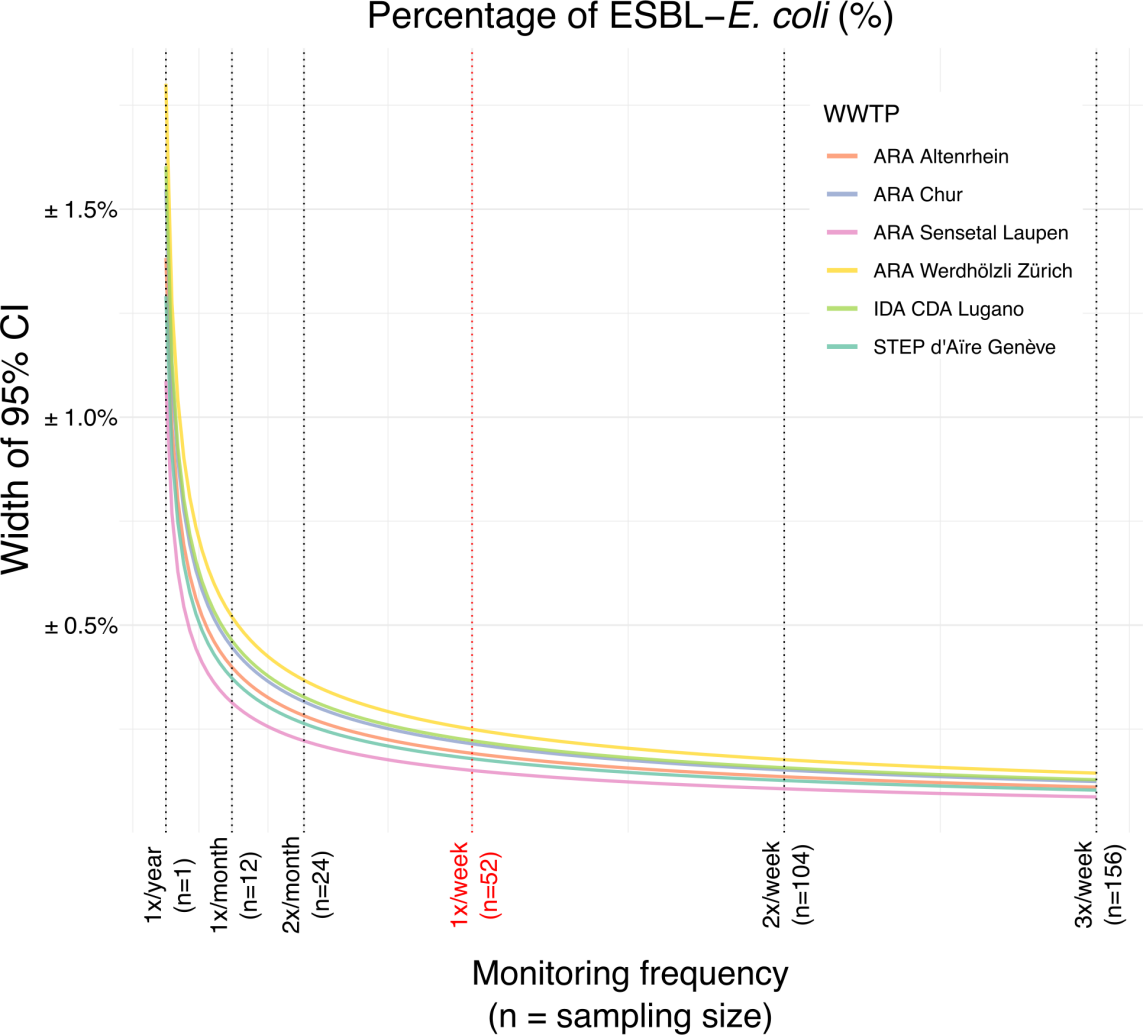
Effect of monitoring frequency on the confidence interval of extended-spectrum β-lactamase-producing *Escherichia coli* percentage in wastewater. CI: confidence interval; *E. coli: Escherichia coli*; ESBL: extended-spectrum β-lactamase; WWTP: wastewater treatment plant. The red vertical line highlights the sampling frequency adopted within our study (once a week).

One year of weekly ESBL-*E. coli* data allowed us to explore the impact of monitoring frequency on uncertainty in the estimated annual mean percentage of ESBL-*E. coli* (Fig. 4). Our analysis, based on the assumption of a consistent annual mean ESBL-*E. coli* percentage, demonstrates that sampling once a week results in a 95% CI width below ±0.25%. Variations exist among WWTPs, notably with Zurich, Lugano, and Chur displaying higher uncertainty in ESBL-*E. coli* percentage compared to other WWTPs at a given sampling frequency.

**Fig 5:**
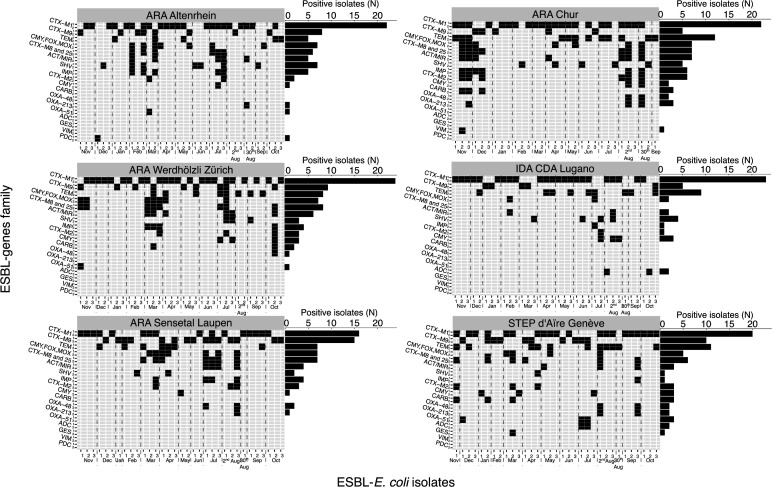
Distribution of 18 extended-spectrum β-lactamase gene families among 201 ESBL-*E. coli* isolates isolated from wastewater, Switzerland, November 2021 to October 2022. *E. coli: Escherichia coli;* ESBL: extended-spectrum β-lactamase; N: number of isolates positive to the gene family. Screening of 234 ESBL-*E. coli* isolates (*x*-axis) for 18 β-lactamase gene families (*y*-axis) using dMLA with a 36-probe pair mixture (two for each family; named 1 and 2 on the *y*-axis). Black rectangles indicate the presence of the ESBL-gene family in the ESBL-*E. coli* isolate. ESBL-*E. coli* isolates are grouped by wastewater treatment plants (WWTPs) and arranged by sampling date (November 2021 to October 2022). Horizontal barplots on the side indicate the number (N) of ESBL-*E.coli* positive to each ESBL-gene family in the respective WWTP. Only strains positive for at least one gene are shown on the heatmap; meaning that both probe-pairs of that gene family were detected in the isolate. Thirty-three strains were negative for all genes and are not represented.

The digital Multiplex Ligation Assay (Fig. 5; Table S7) showed that out of 234 ESBL-*E. coli* isolates, 201 (86%) tested positive for at least one target gene family. The positivity rate was consistent across WWTPs, ranging from 72% (*n* = 31 out of 39) in Chur to 92% (*n* = 36 out of 39) in Altenrhein. The most prevalent ESBL-gene family was CTX-M1, identified in 126 isolates (54% of all screened, 95% CI = 48–59%), present in each WWTP in a minimum of 40% of isolates tested. CTX-M9 and TEM were the second most common, with CTX-M9 detected in 22% (95% CI = 14.6–29.9%) and TEM also detected in 22% (95% CI = 16.9–27.5%) of the total ESBL-*E. coli* isolates screened. Less frequent gene families (GES, VIM, and PDC) were found in only one isolate each in specific WWTPs (0.4% prevalence, 95% CI = 0–1.3%). All 18 targeted ESBL-gene families were detected at least once, but there were no instances where all 18 ESBL-gene families were detected within a single WWTP (Fig. 5). Geneva exhibited the highest number of unique gene families (16 out of 18), and Lugano had the least with 10 gene families. Carbapenem resistance, induced by OXA-48, OXA-51, and OXA-231, was infrequently detected in a small number of isolates from all WWTPs except Lugano. No temporal trends in the distribution of ESBL-gene families among isolates were observed (Fig. S5).

## DISCUSSION

Our results reveal widespread dissemination of ESBL-*E. coli* in Swiss wastewater, with a median concentration of 6.8 × 10^2^ CFUs/mL and corresponding loads of 2.1 × 10^8^ CFUs/(person-day). This aligns with previous concentration estimates in Germany (7.6 × 10^2^ CFUs/mL) (24) and the canton of Basel, Switzerland (9.5 × 10^1^ CFUs/mL) (12). Across the six Swiss WWTPs, we observed a median overall ESBL-*E. coli* percentage of 1.6% (IQR = 0.89), consistent with global rates reported in Sweden (2.3%) (4) and Japan (2.2%) (25). European data ranged from 1.6% in Greece to 4.4% in Germany, with other countries falling in between, including Denmark, Finland, Norway, Belgium, Italy, and Spain (7).

Studies have demonstrated a correlation between antibiotic resistance rates in *E. coli* from wastewater and clinical isolates, with variations in resistance patterns across geographical locations (5, 7). Notably, significant correlations were identified for aminopenicillins and fluoroquinolones, but not specifically for ESBL-producing *E. coli* (7). Our study highlighted significant variations in ESBL-*E. coli* percentages on a spatial scale, indicating potential differences in carriage rates at each location. The larger WWTPs investigated in terms of number of inhabitants connected to the sewage, namely, Zurich, Geneva, and Lugano, showed significantly higher ESBL-*E. coli* percentages throughout the sampling period compared to the smallest WWTPs of Chur and Sensetal-Laupen. Similarly, densely populated regions in Germany also showed increased ESBL-*E. coli* percentage in wastewater (24), suggesting that higher population density facilitates ESBL-*E. coli* transmission.

There are other potential causes for higher percentages of ESBL-*E. coli* than population density alone. Zurich and Geneva are also home to large international airports, associated with increased international travel and food import, which are known risk factors for the spread of resistant bacterial strains across borders (26). Furthermore, the cities of Zurich and Geneva contain multiple healthcare clinics and hospitals, known to have higher carriage rates compared to community settings (23). An alternative explanation could be that individuals from communities contributing to larger wastewater treatment facilities, encompassing broader geographic areas or more densely populated regions, may have higher proportions of ESBL-*E. coli* in their gut flora compared to individuals contributing to smaller facilities.

Infrastructure design of wastewater treatment plants and operational conditions can indirectly affect the percentage of ESBL-*E. coli* by determining wastewater residence time. Residence time might influence selection pressure. However, in our study, we assume that the short maximum residence time of approximately 6 h in Swiss sewage systems minimizes the potential for such selection and proliferation of ESBL-*E. coli*, leading us to consider its effect negligible in our study. Linking wastewater results to human carriage rates also relies on the assumption that ESBL-*E. coli* in wastewater influent from other sources (i.e., animal shedding, agricultural runoff, and combined sewers) is negligible relative to the contributions from people directly.

Wastewater-based estimates of ESBL-*E. coli* percentage may serve as proxies for ESBL-*E. coli* carriage rates within the population. Two data points are missing to validate this assumption for Switzerland: the proportion of ESBL-*E. coli* out of total *E. coli* in Swiss carriers and the prevalence of ESBL-*E. coli* carriage in the gut amongst people in Switzerland. When considering the population-averaged median percentage of ESBL-*E. coli* across all WWTPs of 1.9% across all WWTPs, and assuming an ESBL-*E. coli* carriage prevalence aligns with the European estimate of 6% (22), the proportion of ESBL-*E. coli* out of total *E. coli* in the gut in the Swiss population would be approximately 32%. Alternatively, using an average population-based ESBL-*E. coli* proportion in the gut of 19.3% reported in Bangladesh (23), the ESBL-*E. coli* carriage prevalence in Switzerland ranges between 9.2% and 10.5%, surpassing the European estimate. The discrepancy between the 6% ESBL-*E. coli* carriage prevalence reported in Europe and the estimated 9.2–10.5% in Switzerland emphasizes the complex relationship between wastewater ESBL-*E. coli* percentages and community carriage rates. Directly matching these percentages on a one-to-one basis is challenging due to the complex matrix of wastewater, which is affected by various factors beyond just human waste. Despite these complexities, our analysis supports a link between wastewater metrics and community carriage rates, suggesting that wastewater could serve as a valuable indicator for ESBL-*E. coli* carriage prevalence within the population. However, further cohort studies on prevalence rates and shedding loads in Switzerland are essential to better understand this relationship and to validate the assumptions of our model. These studies would also help understanding whether regions with higher ESBL-*E. coli* percentages in wastewater face an elevated risk of ESBL-*E. coli* infections compared to areas with lower percentages. Such research would offer a comprehensive in understanding of the relationship between ESBL-*E. coli* presence in wastewater and its potential public health implications.

Observed temporal variations in ESBL-*E. coli* concentrations may be influenced by factors like precipitation and water temperature, which impact runoff and bacterial persistence in sewer transport (27). Elevated air temperatures can warm wastewater, promoting bacterial proliferation, but also increase the flow of nutrient-rich runoff from both urban and agricultural landscapes into sewage systems, a phenomenon particularly pronounced during warmer and wetter periods. This dynamic, in combination with seasonal variations in human activities and chemical reactions that are temperature-dependent, can lead to marked fluctuations in the volume of wastewater, its composition, and its microbial load. We found some association between ESBL-*E. coli* percentage and loads and air temperature, but no clear trend emerged. Previous studies showed similar limited climate influence on wastewater measurements, indicating site-specific variability and sporadic positive correlations between outdoor temperature and antibiotic-resistant bacteria concentration (28, 29).

Surveillance of AMR using wastewater will require frequent monitoring. The monitoring frequency should represent an optimization of available resources and the desired level of estimate of precisions. Our analysis reveals that increasing the sampling frequency to at least once per week results in a 95% CI of approximately ±0.25% for ESBL-*E. coli* percentage. Less frequent sampling, such as monthly monitoring, leads to a 95% CI of about ±0.5%. Sampling frequency is therefore an optimization between budget and logistical constraints (necessitating fewer samples) and acceptable uncertainty in the estimated percentage. Nevertheless, our study highlights that even with reduced sampling, reasonably accurate estimates within a broader confidence interval can still be obtained.

A subset of 234 ESBL-*E. coli* isolates from the six wastewater treatment plants were screened for ESBL-gene families using digital multiplex ligation assay. Of these, an ESBL-gene family was detected in 86% (*n* = 201) of them. In the remaining 14% (*n* = 33), no ESBL-gene was identified. This may be because the gene responsible for ESBL resistance was not included among the genes detected by the method (18). Similar findings occurred in a Polish study, suggesting the presence of other genes in the same molecular class A (30). Whole-genome sequencing techniques could offer deeper insights into the observed resistance phenotype.

The most prevalent ESBL-gene family in our study was CTX-M-1, found in 54% of screened ESBL-*E. coli* isolates, followed by CTX-M-9 and TEM, present in 22% of isolates. Similar patterns were observed in European studies in France, the Netherlands, and Poland (16, 30, 31). CTX-M-15, a subtype of CTX-M-1, is widely dominant in Europe and commonly associated with the ST131 clone, known for causing antimicrobial-resistant infections in humans globally (32). This suggests the likely presence of CTX-M enzymes in hospitals or households connected to the investigated wastewater treatment plants.

We found low percentages (2.1–3%) of carbapenem-resistant genes, including OXA-48, OXA-51, and OXA-231. Geneva exhibited all three OXA-like gene families, while Lugano had none, indicating geographically constrained circulation. Despite low prevalence, the presence of OXA-like genes in Switzerland raises concerns due to their increasing incidence in clinical settings (33). VIM and PDC were detected in only 0.4% of screened isolates, aligning with prior observations (34), emphasizing the ability of wastewater monitoring to detect genes with low prevalence and raising questions about their presence in Swiss clinics (35). Sewage analysis, complementing traditional clinical surveillance, offers insights and challenges in detecting rare resistance forms, underscoring its potential and limitations for early warnings (36).

In our study, surveyed sites represented large urban areas, covering 14% of Switzerland’s population. Consequently, the obtained ESBL-*E. coli* data may not reflect the entire country, especially in rural or differently populated regions. Additionally, the study detected ESBL-genes only in 86% of isolates, with potential gaps due to protocol limitations. Whole-genome sequencing could offer insights for the remaining 14%. Species confirmation for ESBL-*E. coli* was not conducted, but the use of CHROMagar with 99.3% specificity for *E. coli* mitigated biases. Notably, the sampling volume of 100 µL for plating may have caused selection bias, for example, the recovery of high abundance isolates, potentially affecting the number of representativeness of isolates. Finally, the 48-h interval between sampling and processing, despite precautions like storage at 4°C and use of ice packs, may have introduced biases due to bacterial growth, decay, or selective pressures, potentially influencing the results and affecting the representation of ESBL-*E. coli* populations.

In conclusion, the widespread prevalence of ESBL-*E. coli* provides a foundational benchmark useful for informing future trends of AMR population-level carriage in Switzerland. It highlights the potential for investigating the factors driving abundance shifts and their applicability in reducing carriage rates. The estimated nationwide ESBL-*E. coli* carriage prevalence, ranging from 9.2% to 10.5%, surpasses the European estimate, possibly due to escalating global antimicrobial resistance rates. However, this conclusion is contingent upon the further validation of the assumptions of our model, including that: (i) there is a one-to-one relationship between wastewater ESBL-*E. coli* levels and population carriage rates, and (ii) the proportion of ESBL-*E. coli* in the gut of Swiss carriers is similar to that observed in Bangladeshi children. Further validation of these assumptions would help refine prevalence estimates. Expanding our research to include other pathogens and cohort studies examining carriage rates in Switzerland would enrich our understanding of AMR dynamics and associated risks for healthcare systems and communities. Furthermore, extending these approaches to communities with decentralized sanitation is needed to gather global insights. Establishing robust connections between our findings and clinical contexts can elucidate the direct impact of AMR gene prevalence on public health. Finally, regular monitoring of antimicrobial-resistant pathogens in wastewater across various communities enables the tracking of population-level trends and changes in resistance, and could be used to inform the impact of public health interventions.

## Data Availability

All data or links to data as well as all scripts are available at our online repository at https://github.com/EawagPHH/esblec-monitor-ww-21-22.git. The sequence data is available at SRA under the BioProject ID PRJNA1092873. Data on precipitation and temperature were obtained from the Federal Office of Meteorology and Climatology MeteoSwiss at https://gate.meteoswiss.ch/idaweb/system/stationList.do (IDAWEB 1.3.5.0 2016 MeteoSwiss).

## SUPPLEMENTAL MATERIAL

Supplemental Material msphere.00760-23-s0001.pdf Tables S1 to S7 and Figures S1 to S5.

ASM does not own the copyrights to Supplemental Material that may be linked to, or accessed through, an article. The authors have granted ASM a non-exclusive, world-wide license to publish the Supplemental Material files. Please contact the corresponding author directly for reuse.
